# Effects of Blanching on Phytochemical Retention, Antioxidant Capacity, and Microstructure of Banana Flower (*Musa* spp., Musaceae)

**DOI:** 10.1002/fsn3.72365

**Published:** 2026-09-11

**Authors:** Saurabh Bhosle, Sonia Morya, Robert Mugabi

**Affiliations:** ^1^ Department of Food Technology and Nutrition, School of Agriculture Lovely Professional University Phagwara India; ^2^ Department of Food Technology and Nutrition, SFTNB, CAES Makerere University Kampala Uganda

**Keywords:** antioxidant activity, bioactive compounds, microstructure, microwave blanching, *Musa* spp., thermal stability

## Abstract

Banana flower (*Musa* spp., Musaceae) is an underutilized agricultural by‐product rich in dietary fiber, phenolic compounds, and antioxidants. However, its rapid deterioration and enzymatic browning may necessitate the implementation of effective pretreatment strategies to facilitate the preservation of its nutritional and functional quality. The present investigation evaluated the potential influence of microwave blanching, citric acid immersion, and hot‐water blanching on the phytochemical profile, antioxidant activity, microstructural characteristics, and thermal properties of banana flower. Banana flowers were subjected to microwave blanching (720 W for 30, 35, and 40 s), citric acid immersion (1%, 3%, and 5%), and hot‐water blanching (100°C for 1, 3, or 5 min). Proximate composition, total phenolic content (TPC), total flavonoid content (TFC), antioxidant activity (DPPH, ABTS, and FRAP assays), scanning electron microscopy (SEM), X‐ray diffraction (XRD), and differential scanning calorimetry (DSC) analyses were performed. Data were analyzed using two‐way ANOVA, and significant differences among treatment means were evaluated using appropriate post hoc tests at *p* < 0.05. Fresh banana flower contained 71.15% moisture, 13.01% crude fiber, 5.58% protein, 4.45% fat, and 3.71% ash, which may indicate substantial nutritional potential. Among all treatments, microwave blanching for 40 s (MW40) exhibited the highest TPC (7.60 mg GAE/g) and TFC (4.90 mg QE/g), alongside elevated antioxidant capacities in DPPH, ABTS, and FRAP assays (*p* < 0.05). SEM analysis revealed extensive pore formation and cell‐wall disruption, while XRD showed a reduction in crystallinity index to approximately 29.7%, which may suggest enhanced amorphization and improved extractability of bound phenolics. DSC analysis demonstrated that MW40 retained considerable thermal stability (Δ*H* = 180.27 J/g) compared with hot‐water‐blanched samples, thereby potentially indicating the preservation of molecular integrity despite structural modification. Optimized microwave blanching (720 W, 40 s) enhanced bioactive compound retention, antioxidant capacity, and extractability while preserving favorable thermal characteristics of banana flower. These findings support its potential as a pretreatment strategy for functional food ingredients; however, further optimization and storage stability studies are required to confirm adequate enzymatic stabilization and industrial applicability. Future studies should investigate storage stability, the bio‐accessibility of bioactive compounds, and industrial‐scale applications of microwave‐blanched banana flower.

## Graphical abstract

Hot‐water blanching (100°C, 1–5 min), citric acid treatment (1%–5% w/v in water), and microwave blanching (720 W, 30–40 s) were employed to modify the bioactive compounds of banana flowers. Microwave blanching for 40 s yielded the highest amounts of total phenolics (7.60 mg GAE/g), total flavonoids (4.90 mg QE/g), and antioxidant activity. The treatment produced a porous and less‐crystalline matrix, which facilitated the release of bioactive compounds and maintained thermal stability. Thus, microwave blanching is a rapid and efficient pretreatment method for the production of a functional food ingredient from banana flowers.

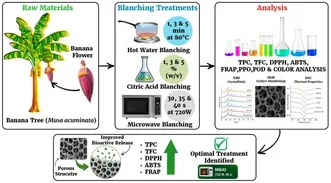

**Abbreviations**
ABTS: 2, 2′‐Azino‐bis (3‐ethylbenzothiazoline‐6‐sulfonic acid)
ANOVA: analysis of variance
BHA: Butylated Hydroxyanisole
BHT: Butylated Hydroxytoluene
C: Control (No blanching)
CA: Citric Acid
CA1%: Citric Acid at 1% concentration
CA3%: Citric Acid at 3% concentration
CA5%: Citric Acid at 5% concentration
CAB: Citric Acid Blanching
DPPH: 2,2‐Diphenyl‐1‐picrylhydrazyl
FRAP: Ferric Reducing Antioxidant Power
GAE: Gallic Acid Equivalent
HSD: Honestly Significant Difference
HW: Hot Water Blanching
HW1: HW3
HW5: Hot Water Blanching for 1, 3, and 5 min
HWB: Hot Water Blanching
mg GAE/g: Milligrams of Gallic Acid Equivalent per gram
mg QE/g: Milligrams of Quercetin Equivalent per gram
mg TE/g: Milligrams of Trolox Equivalent per gram
MW: Microwave Blanching
MW30: MW35
MW40: Microwave Blanching at 720 W for 30, 35, and 40 s
MWB: Microwave Blanching
POD: Peroxidase
PPO: Polyphenol Oxidase
QE: Quercetin Equivalent
SD: Standard Deviation
TE: Trolox Equivalent
TFC: Total Flavonoid Content
TPC: Total Phenolic Content

## 1 Introduction

Banana flowers represent the largest and least exploited by‐product of *Musa* spp. cultivation and are generally discarded as waste, although they are rich in dietary fiber, potassium, magnesium, and plant‐based protein (Senevirathna and Karim 2024). In South Asian and Southeast Asian food cultures, including India, banana flower has a long tradition of culinary and ethnomedicinal use, with all parts of *Musa acuminata* documented across diverse tribal and regional communities (Mathew and Negi 2016). In addition, banana flowers contain a diverse profile of bioactive compounds, mainly polyphenols, that possess high antioxidant, antimicrobial, and anti‐inflammatory activities (Govindaraj 2022), and recent epidemiological and clinical studies have suggested that regular consumption of banana flowers may reduce the risk of cardiovascular disease and other oxidative stress‐mediated disorders (Chowdary et al. 2022; Kumari et al. 2023). However, the primary constraint to the commercial exploitation of banana flowers is their rapid postharvest deterioration, which is caused by the enzymatic oxidation of phenolic compounds by polyphenol oxidase (PPO) and peroxidase (POD), leading to rapid enzymatic browning, and consequently, loss of visual appeal (Kaewjumpol et al. 2021). Although Senevirathna and Karim (2024) provided a comprehensive review of the nutritional and phytochemical profile of *Musa* by‐products broadly, their work did not systematically evaluate processing‐induced changes specific to banana flower, nor did it address the comparative efficacy of different blanching strategies on this tissue.

There is a need for effective stabilization methods to extend shelf life while preserving the nutritional value and functional bioactivity of banana flowers, and this can be achieved through various blanching methods. Blanching is the most common pretreatment (Xiao et al. 2017) used to inactivate browning enzymes and reduce microbial loads in plant materials. Conventional hot‐water blanching (HWB) is the most widely practiced due to its simplicity, but it generally causes leaching of water‐soluble nutrients and thermal degradation of heat‐sensitive antioxidants (Nambi et al. 2016). Chemical blanching with organic acids such as citric acid inhibits enzymatic browning by lowering pH (Nguyen et al. 2019) below the optimal range for PPO activity, although its effectiveness depends on concentration, and it may adversely affect flavor and overall sensory quality. Microwave blanching (MWB) utilizes dielectric heating to rapidly increase tissue temperature, enabling faster enzyme inactivation with reduced processing times compared with conventional methods (Guzik et al. 2021), and has been associated with better retention of structural integrity and phytochemical content (Boateng 2022). While the effects of these blanching methods have been examined in other *Musa* by‐products such as banana peel and pseudostem, studies focusing specifically on banana flower are limited, and no study to date has directly compared thermal, chemical, and microwave‐based pretreatments side by side on this tissue.

However, the relationship between blanching‐induced changes in tissue microstructure and the extractability of bioactive compounds remains a critical area requiring further investigation.

To our knowledge, no study has simultaneously compared hot‐water, citric acid, and microwave blanching on banana flower phytochemistry while integrating scanning electron microscopy (SEM), X‐ray diffraction (XRD), and differential scanning calorimetry (DSC) to mechanistically link matrix‐level structural changes to bioactive compound retention. To address these knowledge gaps, this study systematically examines the effects of hot‐water, citric acid, and microwave blanching on the phytochemical composition and antioxidant activity of banana flowers, using advanced analytical techniques such as SEM, XRD, and DSC to investigate microstructural and thermal changes in the plant matrix during processing. Consequently, this study aims to improve and optimize these stabilization strategies to support the valorization of banana flowers from a low‐value agricultural by‐product to a high‐value functional ingredient with food and nutraceutical applications.

## 2 Materials and Methods

### 2.1 Sample Procurement and Preparation

**FIGURE 1:**
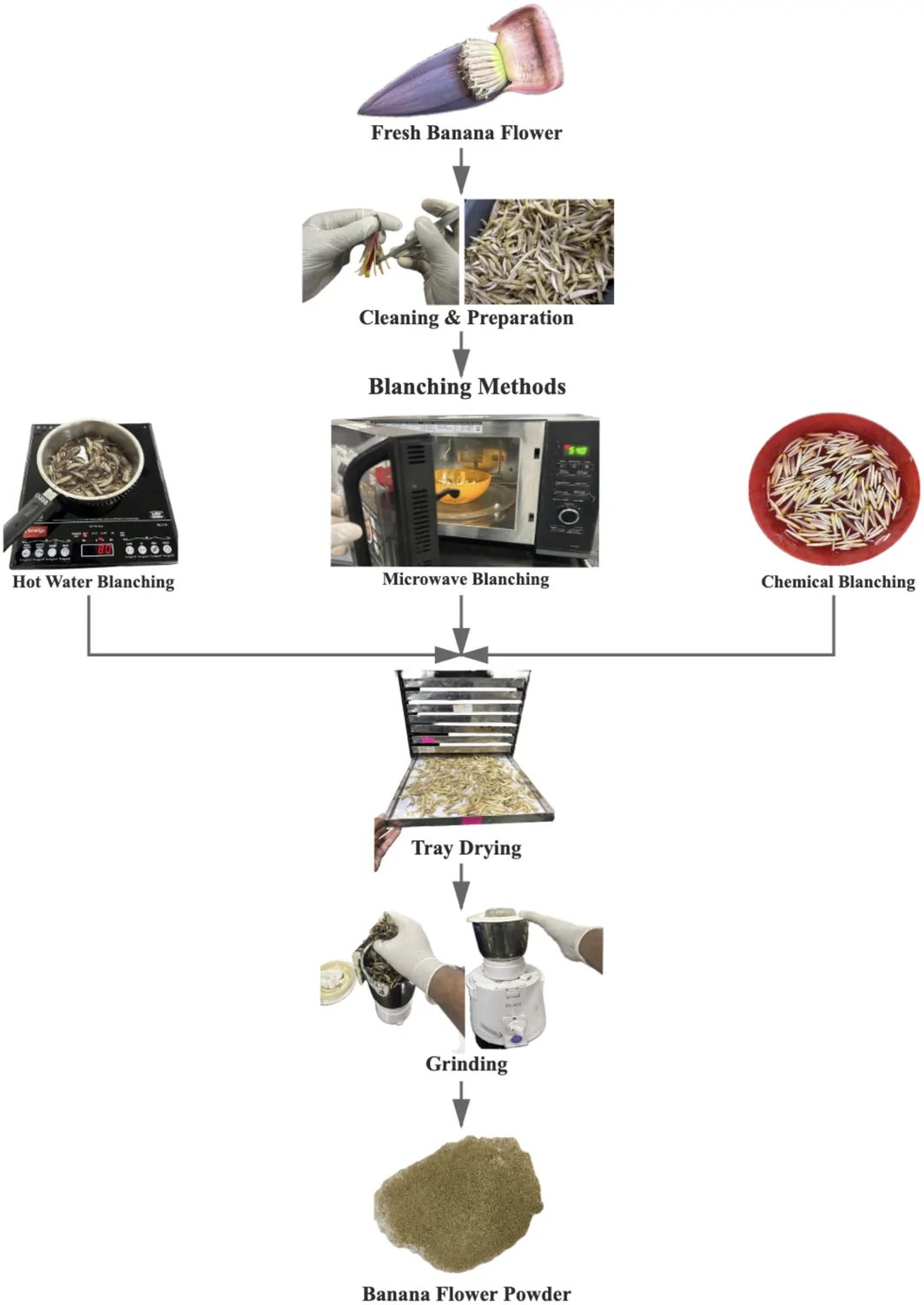
Processing of blanching methods for banana flowers.

*Musa acuminata* Colla (banana) flowers were procured in June 2025 from a local agricultural farm in Kerala, India (GPS coordinates: 8°45′31.6″N 77°02′37.7″ E). Plant material was authenticated by the Botanical Survey of India (BSI), Southern Regional Centre, Coimbatore, Tamil Nadu, India. The authentication certificate was issued under reference number BSI/SRC/5/23/2025–26/Tech./487 dated 10 June 2025. The authenticated specimen was returned for preservation in the institutional herbarium. The separation of the flowers was conducted to remove outer bracts, while rinsing with distilled water facilitated the elimination of surface dust and impurities. The application of distinct blanching treatments (chemical, hot‐water, and microwave) was performed upon the cleaned samples; following immediate cooling, the material was tray‐dried at 50°C until a constant weight was achieved. The dried samples were ground into a fine powder using a laboratory grinder and stored in airtight containers at 4°C for up to 90 days until subsequent analyses, as shown in Figure 1 (processing workflow).

### 2.2 Blanching Treatments

Three blanching techniques were employed. Each method was optimized based on its principal process variable: citric acid (CA) blanching varied chemical concentration at fixed time and temperature; hot‐water (HW) blanching varied immersion time at fixed temperature; and microwave (MW) blanching varied irradiation time at fixed power. The selection of specific levels for each method was guided by preliminary trials and the ranges reported in prior literature (Das and Dhua 2019; Kaewjumpol et al. 2021; Wickramarachchi and Ranamukhaarachchi 2005).

*(1) Citric acid (CA) blanching*: Samples were immersed in citric acid solutions at concentrations of 1%, 3%, and 5% (w/v) prepared in distilled water at ambient temperature (27°C ± 2°C) for 1 min. The selection of this fixed immersion time was predicated on preliminary trials, which suggested that concentration, rather than duration, may represent the dominant variable affecting color retention under ambient‐temperature acid treatment (Sarkar et al. 2024). Following immersion, samples were immediately cooled in an ice‐water bath. *(2) Hot water (HW) blanching*: Samples were immersed in boiling water (100°C) for 1, 3, or 5 min, followed by immediate cooling in an ice‐water bath. Blanching times of 1, 3, and 5 min were selected based on previous studies on *Musa* spp. demonstrating progressive oxidative enzyme inactivation with increasing thermal exposure (Jha et al. 2021). *(3) Microwave (MW) blanching*: Prior to treatment, the initial moisture content of the fresh banana flower samples was determined by oven drying at 105°C according to AOAC Method 934.01. Microwave blanching was performed using a domestic microwave oven (LG Electronics, South Korea; Model MC3286BLU; rated power: 720 W; operating frequency: 2450 MHz). Samples were exposed to microwave treatment for 30, 35, and 40 s. These treatment durations appear justified based on previous studies reporting effective oxidative enzyme control and quality retention following short‐duration microwave blanching in *Musa* spp. and related plant tissues (Hamid et al. 2024). Each sample (~50 g) was placed in a glass dish and subjected to microwave heating. Post‐treatment, samples were immediately cooled in an ice‐water bath.

Control samples, absent of blanching intervention, were maintained at ambient temperature (27°C ± 2°C). Consistent sample weight (~50 g) and cooling conditions (ice‐water bath, 2 min) were maintained across all treatments.

### 2.3 Proximate Composition and Functional Analysis

Proximate composition was determined following the standard methods of the Association of Official Analytical Chemists (AOAC 2019): moisture content (AOAC 934.01; oven drying at 105°C), ash (AOAC 923.03; muffle furnace at 550°C), crude protein (AOAC 960.52; Kjeldahl method, *N* × 6.25), crude fat (AOAC 920.39; Soxhlet extraction), and crude fiber (AOAC 962.09; acid/alkali digestion). Total carbohydrates were calculated by the difference method.

The assessment of functional properties, including water absorption capacity (WAC), oil absorption capacity (OAC), bulk density, water solubility index (WSI), and swelling power (SP) was conducted in accordance with standardized procedures frequently applied to plant‐derived powders and food ingredients, incorporating minor modifications where deemed necessary (Senevirathna et al. 2024; Tran et al. 2026). Emulsifying capacity (EC) was determined by homogenizing 1 g of banana flower powder with 10 mL of distilled water and 10 mL of vegetable oil at 10,000 rpm for 1 min using a high‐speed homogenizer. The emulsion was centrifuged at 1200 × g for 5 min, and EC was calculated as the ratio of the emulsified layer height to the total mixture height, expressed as a percentage. Foaming capacity (FC) was determined by homogenizing 1 g of powder in 50 mL of distilled water at 10,000 rpm for 2 min. The volume before and after whipping was recorded, and FC was expressed as the percentage increase in volume after whipping (Weyya et al. 2025).

### 2.4 Phytochemical and Antioxidant Assays

The extraction of phenolic compounds was performed by combining 0.5 g of banana flower powder with 10 mL of 80% (v/v) methanol, shaking at 30°C for 2 h, and centrifuging at 5000 × *g* for 10 min. This extraction cycle was repeated three times; the combined supernatants were pooled and made up to a fixed volume before analysis. The resulting supernatant was analyzed for (1) TPC, determined using the Folin–Ciocalteu colorimetric method, with slight modifications to the procedure described by Singleton and Rossi (1965). The absorbance was measured by spectrophotometry, and gallic acid was used as the calibration standard. Results were expressed as milligrams of gallic acid equivalents per gram of sample (mg GAE/g). (2) TFC was determined using the aluminum chloride colorimetric method, with slight modifications to the procedure reported by Zhishen et al. (1999). Quercetin was used as the calibration standard, and the results were expressed as milligrams of quercetin equivalents per gram of sample (mg QE/g). (3) Antioxidant activity was evaluated using three complementary assays. DPPH radical‐scavenging activity was determined following the method of Brand‐Williams et al. (1995). ABTS radical cation‐scavenging activity was determined according to Re et al. (1999), while ferric reducing antioxidant power (FRAP) was assessed following the method of Benzie and Strain (1996). Results were expressed as milligrams or micromoles of Trolox equivalents per gram of sample (TE/g), as appropriate to the corresponding assay.

### 2.5 Enzyme Activity Assays

The activities of PPO and POD were determined immediately after each blanching treatment and cooling. Fresh banana flower tissue (1 g) was homogenized with 5 mL of 0.1 M sodium phosphate buffer (pH 6.8) at 4°C using a chilled mortar and pestle. The homogenate was centrifuged at 10,000 × *g* for 20 min at 4°C, and the resulting supernatant was used as the crude enzyme extract. All enzyme activity assays were performed in triplicate at 25°C ± 2°C. One unit (U) of enzyme activity was defined as the amount of enzyme causing a change in absorbance of 0.001 min^−1^ g^−1^ fresh weight (FW).

#### 2.5.1 Polyphenol Oxidase (PPO) Activity

Polyphenol oxidase activity was determined according to the method of Kaewjumpol et al. (2021) with minor modifications. The reaction mixture consisted of 2.8 mL of 0.1 M catechol prepared in 0.1 M sodium phosphate buffer (pH 6.8) and 0.2 mL of crude enzyme extract. The increase in absorbance at 420 nm, resulting from the oxidation of catechol, was monitored every 30 s for 3 min using a UV–Vis spectrophotometer. PPO activity was expressed as U g^−1^ FW. Residual PPO activity (%) was calculated using the following equation:

$$\text{Residual}\;\mathrm{PPO}\;\text{activity}\;\left(\%\right)=\frac{\mathrm{PPO}\;\text{activity of blanched sample}}{\mathrm{PPO}\;\text{activity of unblanched sample}}\times 100$$

#### 2.5.2 Peroxidase (POD) Activity

Peroxidase activity was determined using the guaiacol oxidation method described by Hamid et al. (2024). The reaction mixture contained 2.5 mL of 0.1 M sodium phosphate buffer (pH 6.8), 0.1 mL of 1% (v/v) guaiacol, 0.1 mL of 0.3% (v/v) hydrogen peroxide (H_2_O_2_), and 0.3 mL of crude enzyme extract. The increase in absorbance at 470 nm due to the formation of tetraguaiacol was recorded every 30 s for 3 min using a UV–Vis spectrophotometer. POD activity was expressed as U g^−1^ FW. Residual POD activity (%) was calculated as:

$$\text{Residual}\;\mathrm{POD}\;\text{activity}\;\left(\%\right)=\frac{\mathrm{POD}\;\text{activity of blanched sample}}{\mathrm{POD}\;\text{activity of unblanched sample}}\times 100$$

### 2.6 Color Measurement

Color was measured in blanched and control banana flower powders using a Konica Minolta CR‐400 colorimeter (Konica Minolta Inc., Tokyo, Japan), which was calibrated against a standard white tile prior to measurement. Measurements were recorded in the CIELAB color space under illuminant D65 using a 10° standard observer angle. The color parameters L*, a*, and b* were recorded, representing lightness, redness/greenness, and yellowness/blueness, respectively. Three readings per sample were taken at different positions and averaged. The CIELAB parameters recorded were: L* (lightness, 0 = black to 100 = white), a* (green (−) to red (+)), and b* (blue (−) to yellow (+)).

The total color difference (Δ*E*) from the unblanched control was calculated using the CIE76 formula:

$$\Delta E=\left[{\left(\Delta L*\right)}^{2}+{\left(\Delta a*\right)}^{2}+{\left(\Delta b*\right)}^{2}\right]½$$

where ΔL*, Δa*, and Δb* represent the respective chromaticity variances between the treated sample and the control, a Δ*E* value below one may be considered imperceptible to the human observer; conversely, values between one and 3.3 suggest slight perceptibility, whereas values exceeding 3.3 are typically categorized as clearly perceptible (Sakiroff et al. 2022).

### 2.7 Advanced Structural Characterization

To investigate the mechanistic impact of blanching on the tissue matrix, the following instrumental analyses were performed. (1) SEM: Surface morphology, porosity, and cellular integrity were examined using a JEOL JSM‐7610F Plus field‐emission SEM (JEOL Ltd., Tokyo, Japan) coupled with an Oxford Instruments EDS LN2‐free detector (Oxford Instruments, Abingdon, UK). To prepare for imaging, samples were mounted on aluminum stubs and sputter‐coated with a gold–palladium alloy to a thickness of approximately 10 nm. Images were subsequently acquired at an accelerating voltage of 10 kV and a working distance of 10 mm, with magnifications set at ×500 and ×1000. (2) XRD: Crystallinity indices and structural modifications were analyzed using a Bruker D8 Advance X‐ray diffractometer (Bruker AXS GmbH, Karlsruhe, Germany) with Cu Kα radiation (λ = 0.1541 nm) operating at 40 kV and 40 mA. Diffractograms were recorded over a 2θ range of 5°–80° with a step size of 0.02° and a scan speed of 2° min^−1^. The Crystallinity Index (CrI) was calculated using the Segal empirical method: CrI (%) = [(I₀₀_2_ − Iₐₐ)/I₀₀_2_] × 100, where I₀₀_2_ is the intensity of the crystalline peak at 2θ ≈22.6° and Iₐₐ is the intensity of the amorphous background at 2θ ≈18° (Pereira et al. 2022). (3) DSC: Thermal stability and enthalpy changes (ΔH) were evaluated using a PerkinElmer DSC 6000 (PerkinElmer Inc., Waltham, MA, USA) DSC instrument. Approximately 5–7 mg of each sample was sealed in an aluminum pan, with an empty aluminum pan used as the reference. Samples were heated from 25°C to 300°C at a rate of 10°C/min under a nitrogen purge flow of 50 mL/min.

### 2.8 Statistical Analysis

All analyses were performed in triplicate, and data are presented as means ± standard deviations (SDs). A two‐way analysis of variance (ANOVA) was performed to assess significant differences among treatments, with blanching method type (citric acid, hot water, microwave, and control) as Factor A and treatment level within each method (concentration for CA; time for HW and MW) as Factor B. Where significant differences were detected (*p* < 0.05), differences among treatment means were evaluated using Duncan's multiple range test. For enzyme activity and color parameters, Tukey's honestly significant difference (HSD) test was used for pairwise mean comparisons at *p* < 0.05. All statistical analyses were performed using SPSS Statistics 25.0 (IBM Corp., Armonk, NY, USA).

## 3 Results and Discussion

### 3.1 Proximate and Functional Characterization of Banana Flower

#### 3.1.1 Proximate Composition of Fresh Banana Flower

The proximate analysis of fresh banana flowers (*Musa* spp.) confirms a nutritionally rich composition with considerable potential for functional food applications, as detailed in Table 1. The fresh‐weight moisture content was determined to be 71.15% ± 0.48% (w/w), which is lower than the commonly reported range of 88%–93% for fresh banana floral tissue (Thagunna 2023). This discrepancy likely reflects postharvest wilting during storage and transport prior to analysis, partial dehydration of outer bracts during ambient air exposure, and/or varietal differences in water‐holding capacity specific to the *Musa acuminata* cultivar used in this study. Importantly, this moisture level, while atypical relative to literature means, is consistent with moisture loss observed in commercially handled banana inflorescences and does not necessarily indicate measurement error. Future studies should standardize the time interval between harvest and proximate analysis to reduce this source of variability.

**TABLE 1:** Proximate analysis of banana flower powder.

Parameter	Value % (mean ± SD)
Moisture	71.15 ± 0.48
Fat	4.45 ± 0.10
Fiber	13.01 ± 0.47
Protein	5.58 ± 0.16
Ash	3.71 ± 0.17
Carbohydrates	2.10 ± 0.05

*Note:* Values are expressed as mean ± standard deviation (*n* = 3). All values on a fresh‐weight basis.

Banana flower was a rich source of dietary fiber, with a crude fiber content of 13.01 ± 0.47 g/100 g (% w/w), comparable to reported high‐fiber plant by‐products. The crude protein content was 5.58% ± 0.16% (w/w), confirming its suitability as a plant‐based protein source (literature range: 5%–10%). Ash content was 3.71 ± 0.17 g/100 g (% w/w), reflecting a significant mineral fraction consistent with mineral‐rich floral profiles. Moderate crude fat (4.45% ± 0.10% w/w) and low carbohydrate levels (2.10% ± 0.05% w/w) were also recorded. These findings broadly agree with existing literature and confirm the nutritional potential of underutilized banana floral tissue (Silva et al. 2025; Thagunna 2023).

#### 3.1.2 Functional Properties of Banana Flower Powder

The functional characteristics of banana flower powder are summarized in Table 2. Water absorption capacity (WAC) was 2.45 ± 0.10 g/g, a value substantially influenced by the high fiber content, enabling efficient water binding and texture enhancement in dough, batter, and restructured meat formulations (Ayo‐Omogie 2023). Oil absorption capacity (OAC) was 1.92 ± 0.08 g/g; this parameter supports flavor retention, lubricity, and mouthfeel in high‐fat food systems, consistent with the fat content of the powder (Asouzu et al. 2020). The swelling index was 6.30 ± 0.20 mL/g and solubility was 12.8% ± 0.5% (w/v), indicating good hydration and dispersibility relevant to thermal processing and stability in soups, sauces, and beverages (Dibakoane et al. 2025). Emulsifying capacity was 48.5% ± 1.4% and foaming capacity was 21.6% ± 1.1%, suggesting potential for stabilizing emulsions in mayonnaise, creams, and aerated food systems such as whipped toppings (Weyya et al. 2025). Collectively, these functional properties position banana flower powder as a viable, clean‐label functional ingredient, comparing favorably with other underutilized plant by‐products.

**TABLE 2:** Functional properties of banana flower powder.

Property	Value (mean ± SD)
Water absorption capacity	2.45 ± 0.10
Oil absorption capacity	1.92 ± 0.08
Swelling index (mL/g)	6.30 ± 0.20
Solubility (%)	12.8 ± 0.5
Emulsifying capacity (%)	48.5 ± 1.4
Foaming capacity (%)	21.6 ± 1.1

*Note:* Data are presented as the mean ± standard deviation for the functional parameters of the banana flower (*n* = 3).

### 3.2 Effect of Blanching Treatments on Phytochemical and Antioxidant Properties

Blanching of banana flower extracts under varying conditions significantly influences phytochemical composition and antioxidant capacity, with microwave blanching demonstrating superior retention and enhanced bioactivity compared with hot‐water or citric acid treatments. The subsequent subsections delineate the impact of each treatment on TPC, the TFC, and the antioxidant capacity (DPPH, ABTS, and FRAP).

#### 3.2.1 Effect on Total Phenolic Content

Total phenolic content (TPC) varied significantly (*p* < 0.05) across blanching treatments (Table 3), with MW40 yielding the highest value (7.60 mg GAE/g), followed by MW35 and MW30. Microwave‐treated samples consistently exhibited higher phenolic retention than the control and other pretreatments, indicating that microwave blanching not only arrests enzymatic phenolic degradation but also enhances extractability. The observed increase in TPC is likely attributable to cell‐wall disruption and destabilization of phenolic polysaccharide complexes, which may facilitate the release of cell‐wall‐bound phenolics (Tigreros et al. 2024). The absence of an aqueous phase during microwave treatment further reduces leaching losses (Puligundla 2013). Hot‐water blanching produced the greatest reduction in TPC, with HW5 recording the lowest value (5.30 mg GAE/g), attributable to diffusion of soluble phenolics into the blanching medium and thermal oxidation with extended exposure. Citric acid blanching caused concentration‐dependent TPC reduction, with CA3 and CA5 showing the greatest losses through acid‐mediated solubilization and hydrolytic degradation.

**TABLE 3:** Effect of different blanching methods on antioxidant activity (DPPH, ABTS, FRAP) and phytochemical content (TPC, TFC) of banana flower.

Sample	DPPH (μmol TE/g)	ABTS (μmol TE/g)	FRAP (μmol TE/g)	TPC (mg GAE/g)	TFC (mg QE/g)
C	55.50 ± 1.53ᵇ	60.48 ± 0.40ᵇ	69.42 ± 2.91ᶜ	6.60 ± 0.23ᶜ	4.23 ± 0.12ᶜ
CA1	53.47 ± 0.67ᶜ	59.10 ± 0.60ᶜ	66.93 ± 1.73ᶜ	6.61 ± 0.19ᶜ	4.08 ± 0.10ᶜ
CA3	50.62 ± 2.72ᵈ	52.10 ± 1.82ᵉ	63.45 ± 2.20ᵈ	5.91 ± 0.49ᵈ	3.82 ± 0.07ᵈ
CA5	49.36 ± 0.91ᵈ	54.22 ± 0.35ᵈ	60.90 ± 1.48ᵈ	5.80 ± 0.12ᵈᵉ	3.89 ± 0.10ᵈ
MW30	57.26 ± 1.17ᵃ	60.47 ± 0.22ᵇ	73.06 ± 1.84ᵇ	7.12 ± 0.07ᵇ	4.52 ± 0.03ᵇ
MW35	57.61 ± 0.87ᵃ	63.71 ± 0.85ᵃ	73.82 ± 0.03ᵇ	7.30 ± 0.14ᵇ	4.83 ± 0.20ᵃ
MW40	57.97 ± 1.10ᵃ	63.83 ± 0.74ᵃ	75.72 ± 0.55ᵃ	7.60 ± 0.10ᵃ	4.90 ± 0.03ᵃ
HW1	47.54 ± 4.10ᵈ	54.89 ± 4.51ᵈ	62.07 ± 4.48ᵈ	5.89 ± 0.41ᵈ	3.82 ± 0.17ᵈ
HW3	41.47 ± 0.91ᶠ	52.74 ± 1.44ᵉ	59.83 ± 1.20ᵉ	5.59 ± 0.34ᵉ	3.71 ± 0.04ᵉ
HW5	44.54 ± 1.05ᵉ	50.20 ± 0.28ᶠ	57.93 ± 0.45ᵉ	5.30 ± 0.16ᶠ	3.37 ± 0.20ᶠ

*Note:* Statistical analysis was performed using two‐way ANOVA, and significant differences among treatment means were determined using Duncan's multiple range test (*p* < 0.05). Different superscript letters within a column indicate significant differences among the different blanching methods (*p* < 0.05, Tukey's HSD test).

#### 3.2.2 Effect on Total Flavonoid Content

Total flavonoid content (TFC) followed a trend consistent with TPC (Table 3). The highest flavonoid concentrations were observed in MW40 (4.90 mg QE/g) and MW35 (4.83 mg QE/g), confirming the superior protective effect of microwave blanching on flavonoid integrity. Hot‐water blanching caused the greatest flavonoid losses, with HW5 recording the lowest TFC (3.37 mg QE/g), attributable to leaching and thermal degradation of flavonoid glycosides. Citric acid blanching reduced flavonoid levels at higher concentrations, presumably through acid‐catalyzed cleavage of glycosidic bonds.

The relatively greater flavonoid retention under microwave blanching than under hot‐water blanching is explained by the reduced aqueous exposure during microwave treatment. Flavonoid glycosides are inherently water‐soluble and thermolabile, making them highly susceptible to leaching and thermal degradation in hot‐water systems. Microwave blanching, by achieving rapid internal heating with minimal added water, limits these losses and preserves flavonoid integrity more effectively (Dahiya et al. 2023).

#### 3.2.3 Effect of Blanching Treatments on Antioxidant Activity

Table 3 summarizes the effects of blanching pretreatments on DPPH, ABTS, and FRAP antioxidant activity. Significant differences (*p* < 0.05) were observed across all three assays. Microwave blanching consistently produced the highest antioxidant values. MW40 exhibited the highest DPPH scavenging activity (57.97 μmol TE/g), ABTS radical scavenging activity (63.83 μmol TE/g), and ferric reducing power (FRAP: 75.72 μmol TE/g).

Notably, while TPC showed a statistically significant dose–response trend among microwave treatments (MW40 > MW35 > MW30), DPPH scavenging activity was statistically equivalent across all three microwave durations (MW30: 57.26, MW35: 57.61, MW40: 57.97 μmol TE/g; all sharing the superscript “a” in Table 3). This divergence is biologically plausible: TPC is a quantitative measure of phenolic extractability, whereas DPPH scavenging reflects the collective radical‐quenching capacity of all antioxidant species present. Once enzyme inactivation is sufficient at 30 s to prevent oxidative degradation, incremental gains in extractable phenolics between MW30 and MW40 may not translate to statistically detectable differences in radical scavenging capacity within the sensitivity range of the DPPH assay. This also suggests that antioxidant activity may be substrate‐saturated under MW30 conditions, with further phenolic release beyond this threshold contributing minimally to DPPH reduction.

Hot‐water blanching caused progressive declines in all antioxidant parameters with increasing duration. HW5 recorded the lowest values across DPPH (44.54 μmol TE/g), ABTS (50.20 μmol TE/g), and FRAP (57.93 μmol TE/g), attributable to leaching of water‐soluble antioxidants and heat‐induced degradation of phenolic compounds (Silva et al. 2025). Citric acid blanching produced intermediate antioxidant values, with CA1 demonstrating the best retention among acid treatments, while CA3 and CA5 showed progressive losses likely due to acid‐driven solubilization and hydrolysis of phenolic components (Gil‐Martín et al. 2021).

#### 3.2.4 Polyphenol Oxidase (PPO) and Peroxidase (POD) Activity

**FIGURE 2:**
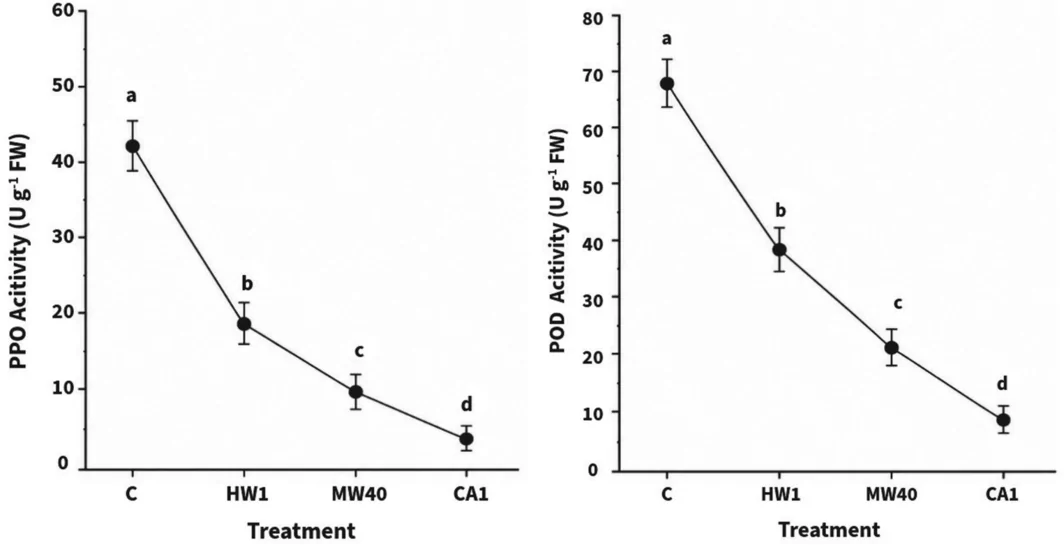
The influence of blanching treatments upon the enzymatic activity of polyphenol oxidase (PPO) and peroxidase (POD) within the banana flower.

All blanching treatments significantly reduced both PPO and POD activity compared with the untreated control (*p* < 0.05), confirming effective enzyme inactivation across treatment types (Table 4; Figure 2). Control samples exhibited the highest PPO activity (42.37 ± 1.84 U g^−1^ FW) and POD activity (68.53 ± 2.16 U g^−1^ FW). Hot‐water blanching reduced PPO to 18.62 ± 0.97 U g^−1^ FW (43.95% residual activity) and POD to 38.74 ± 1.52 U g^−1^ FW (56.53% residual activity). Microwave blanching achieved greater inactivation, reducing PPO to 9.14 ± 0.63 U g^−1^ FW (21.57% residual) and POD to 21.09 ± 1.18 U g^−1^ FW (30.78% residual). Citric acid blanching (1%) produced the most effective enzyme inactivation, reducing PPO to 3.28 ± 0.41 U g^−1^ FW (7.74% residual) and POD to 8.46 ± 0.73 U g^−1^ FW (12.35% residual), consistent with the pH‐dependent denaturation of metalloenzymes under acidic conditions (Lee et al. 2025; Harshitha et al. 2025).

**TABLE 4:** Effect of blanching treatments on polyphenol oxidase (PPO) and peroxidase (POD) activities of banana flower.

Treatment	PPO activity (U g^−1^ FW)	PPO residual activity (%)	POD activity (U g^−1^ FW)	POD residual activity (%)
Control	42.37 ± 1.84ᵃ	100.00	68.53 ± 2.16ᵃ	100.00
HW	18.62 ± 0.97ᵇ	43.95	38.74 ± 1.52ᵇ	56.53
MW	9.14 ± 0.63ᶜ	21.57	21.09 ± 1.18ᶜ	30.78
CA (1%)	3.28 ± 0.41ᵈ	7.74	8.46 ± 0.73ᵈ	12.35

*Note:* Values are expressed as mean ± standard deviation (*n* = 3). Different superscript letters within each activity column indicate significant differences among treatments according to Tukey's HSD test (*p* < 0.05).

Abbreviations: CA, 1% citric acid blanching; FW, fresh weight; HW, hot water blanching; MW, microwave blanching.

The inverse relationship between enzyme inactivation efficiency and phenolic/antioxidant retention across treatments warrants mechanistic discussion. Citric acid achieved the most complete enzyme inactivation yet yielded intermediate TPC and antioxidant values, whereas microwave blanching achieved moderate enzyme inactivation but superior phenolic retention. This apparent paradox is explained by the competing effects of leaching and enzyme‐driven oxidation. In hot‐water blanching, residual enzyme activity (43.95% PPO, 56.53% POD) combined with prolonged aqueous exposure drives oxidative degradation and solute leaching simultaneously, producing the greatest net loss of phenolics. In citric acid blanching, effective enzyme inactivation is offset by acid‐induced solubilization and hydrolytic degradation of phenolic compounds at higher acid concentrations. Microwave blanching achieves a functionally adequate degree of enzyme inactivation sufficient to arrest enzymatic browning within a short treatment time and without an aqueous medium, thereby preserving phenolic compounds from both enzymatic oxidation and leaching. These enzyme activity data directly support the mechanistic interpretation offered in Sections 3.2.1, 3.2.2, 3.2.3 and suggest that microwave blanching is an effective pretreatment for bioactive preservation in banana flower.

#### 3.2.5 CIELAB Color Analysis and Browning/Whiteness Indices

Blanching treatments revealed to induced significant and treatment‐dependent variations in the chromatic parameters of banana flower powder, as summarized in Table 5. The untreated control exhibited the highest L* value (71.34 ± 1.23), indicating the lightest baseline color, along with positive a* (2.17 ± 0.18, slight redness) and b* (22.46 ± 0.94, strong yellowness). Browning Index (BI) was highest in the control (68.42 ± 2.11) and Whiteness Index (WI) was 52.18 ± 1.47, reflecting the characteristic yellow‐brown pigmentation of fresh banana floral tissue. Hot‐water blanching induced the most pronounced color disruption, with the lowest L* (63.82 ± 1.41), a shift to negative a* (−1.34 ± 0.22, greenish hue), the highest total color difference (Δ*E* = 9.74 ± 0.63), and the lowest BI (49.37 ± 1.86) and WI (48.63 ± 1.32). The large ΔE value suggests a perceptible and potentially commercially significant color shift from the control, likely due to chlorophyll degradation, Maillard reactions under prolonged heat exposure, and leaching of pigment compounds into the blanching water. Microwave blanching produced moderate color change (Δ*E* = 6.12 ± 0.54), with L* of 66.57 ± 1.18, slightly negative a* (−0.76 ± 0.19), and reduced BI (54.68 ± 1.74), indicating partial color alteration but better preservation of lightness than with hot‐water treatment. Citric acid blanching (1%) exhibited the least color deviation from the control, with the smallest ΔE (2.83 ± 0.41), highest L* among blanched samples (69.11 ± 1.09), near‐neutral a* (0.48 ± 0.14), and the best‐preserved BI (61.29 ± 1.63) and WI (51.47 ± 1.26) among all blanching treatments. The low Δ*E* and higher BI/WI values for CA1 are consistent with its effective enzyme inactivation (PPO: 7.74% residual; POD: 12.35% residual), which suppresses enzymatic browning, and with the acidic pH environment, which stabilizes anthocyanins and other chromogenic compounds. These color data are consumer‐relevant: a Δ*E* > 3 is generally considered perceptible to the human eye (Haeghen et al. 2000), suggesting that hot‐water‐blanched banana flower might appear noticeably discolored compared to fresh tissue, while citric acid treatment preserves color most acceptably for potential functional food applications.

**TABLE 5:** Evaluation of the potential influence of distinct blanching treatments on the colorimetric parameters of the banana flower.

Treatment	L*	a*	b*	Δ*E*	BI	WI
Control	71.34 ± 1.23ᵃ	2.17 ± 0.18ᵃ	22.46 ± 0.94ᵃ	—	68.42 ± 2.11ᵃ	52.18 ± 1.47ᵃ
HW1	63.82 ± 1.41ᵇ	−1.34 ± 0.22ᵇ	17.83 ± 0.87ᵇ	9.74 ± 0.63ᵃ	49.37 ± 1.86ᵇ	48.63 ± 1.32ᵇ
MW40	66.57 ± 1.18ᶜ	−0.76 ± 0.19ᶜ	19.21 ± 0.72ᶜ	6.12 ± 0.54ᵇ	54.68 ± 1.74ᶜ	50.14 ± 1.19ᶜ
CA1	69.11 ± 1.09ᵈ	0.48 ± 0.14ᵈ	20.87 ± 0.81ᵈ	2.83 ± 0.41ᶜ	61.29 ± 1.63ᵈ	51.47 ± 1.26ᵈ

*Note:* Mean ± SD, *n* = 3. Different superscript letters within a column indicate significant differences (*p* < 0.05, Tukey's HSD test). Δ*E* not applicable for control.

Abbreviations: ΔE, total color difference from control; BI, Browning Index; WI, Whiteness Index.

### 3.3 Advanced Structural Characterization

#### 3.3.1 Scanning Electron Microscopy (SEM)

##### 3.3.1.1 Scanning Electron Microscopic Analysis of Unblanched Banana Flower (Control)

**FIGURE 3:**
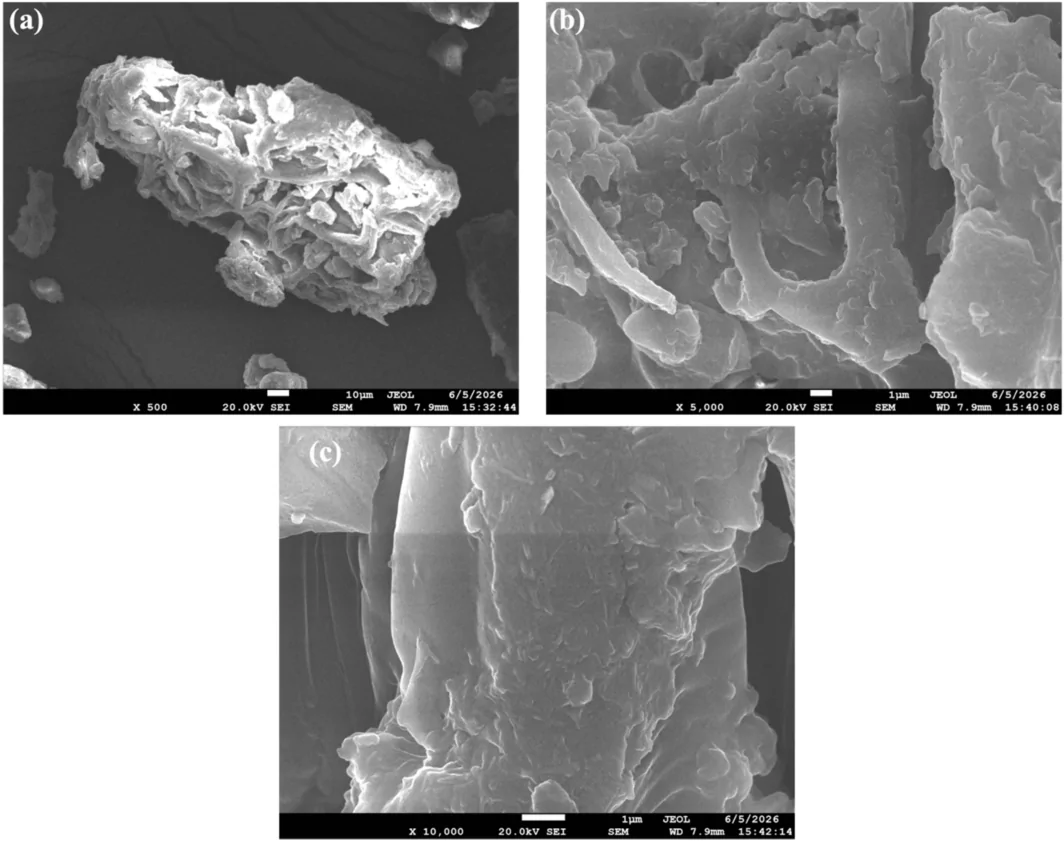
Scanning electron microscopy (SEM) images of an untreated (control) banana flower at (a) 500×, (b) 5000×, and (c) 10,000× magnification, showing the intact cellular architecture with compact tissue organization, well‐defined cell walls, and minimal structural disruption.

The SEM micrographs of the unblanched control banana flower powder appeared to reveal a well‐organized, structurally intact tissue architecture across all magnifications (Figure 3). At 500×, the tissue presented as a dense, compact, and cohesive particle with a continuous, rough surface and no visible pores, fractures, or cellular disruption. The intact surface morphology reflects the unmodified native cell wall matrix, in which cellulose microfibrils, hemicellulose, and pectin remain fully cross‐linked and structurally integrated, providing a physically dense barrier to solvent penetration and bioactive extraction (Holland et al. 2020; Sivan et al. 2023) At 5000×, tightly associated lamellar cell wall layers were evident, with no intercellular separation, void formation, or delamination. The fibrous sheet‐like structures observed at this magnification correspond to intact cell wall lamellae in close contact, consistent with the unmodified middle lamella holding adjacent cells together through Ca^2+^‐mediated pectin cross‐linking (Daher and Braybrook 2015; Peaucelle et al. 2012) At 10,000×, the dominant structures presented smooth to slightly corrugated surfaces with a continuous, dense cell wall matrix and no micropores, exfoliated fragments, or exposed inner wall components. The absence of micro‐fractures or fibril disruption suggests that the enzymatic activity of PPO and POD may have remained largely active in the control, and that phenolic compounds were tightly compartmentalized within intact vacuoles and cell wall–polysaccharide complexes, limiting their extractability. These structural features of the control provide the baseline against which blanching‐induced microstructural modifications in CA1‐, HW1‐, and MW40‐treated samples (Sections 3.3.1.1, 3.3.1.2, 3.3.1.3) can be interpreted. The progressive increase in porosity, delamination, and surface disruption observed across citric acid, hot water, and microwave treatments, relative to this intact control, may elucidate the corresponding variations in TPC, TFC, and antioxidant activity reported in Sections 3.2.1, 3.2.2, 3.2.3.

##### 3.3.1.2 SEM of Banana Flower Blanched With 1% Citric Acid

**FIGURE 4:**
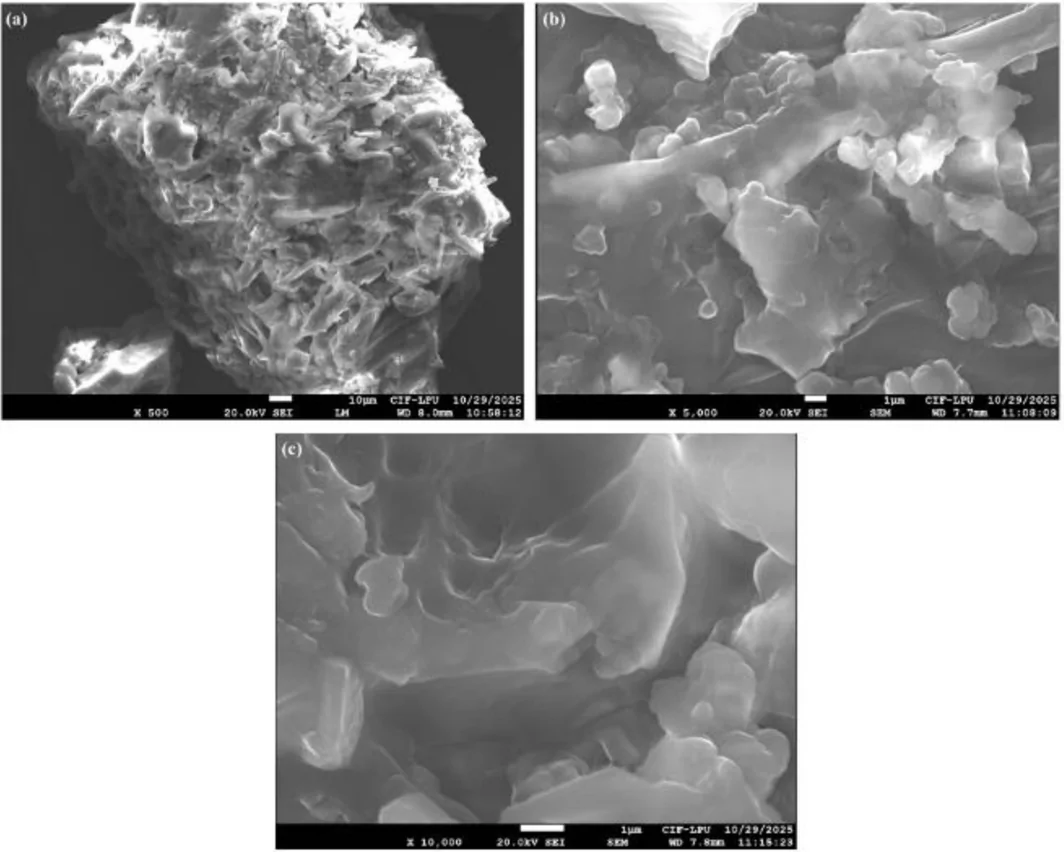
SEM images of banana flower blanched with 1% citric acid at (a) 500×, (b) 5000×, and (c) 10,000× magnification, showing progressive microstructural modification without gross cellular disruption.

SEM images of banana flower blanched with 1% citric acid showed controlled microstructural modification across magnifications (Figure 4). At 500×, the tissue exhibited a smooth, compact, and cohesive surface, indicating that mild acid blanching did not cause gross disruption of cellular integrity, thereby limiting oxidative and leaching losses of bioactive compounds. At 5000×, small fissures and intercellular gaps were evident, reflecting partial weakening of the middle lamella and pectic matrix. Citric acid selectively solubilized pectins and interfered with Ca^2+^ cross‐linking, producing controlled loosening of adjacent cells. These microstructural apertures facilitate mass transfer during extraction. At 10,000×, micropores and slight surface etching were visible, indicating selective removal of amorphous polysaccharides (hemicellulose, pectin) while the cellulose framework remained intact. This porous structure increases specific surface area and accessibility of bound phenolics. Overall, 1% citric acid blanching achieves an optimal balance between tissue softening and structural preservation, mechanistically explaining the high antioxidant activity and phenolic recovery observed for CA1‐treated samples (Malakar et al. 2025).

##### 3.3.1.3 SEM of Banana Flower Blanched With Hot Water (1 Min)

**FIGURE 5:**
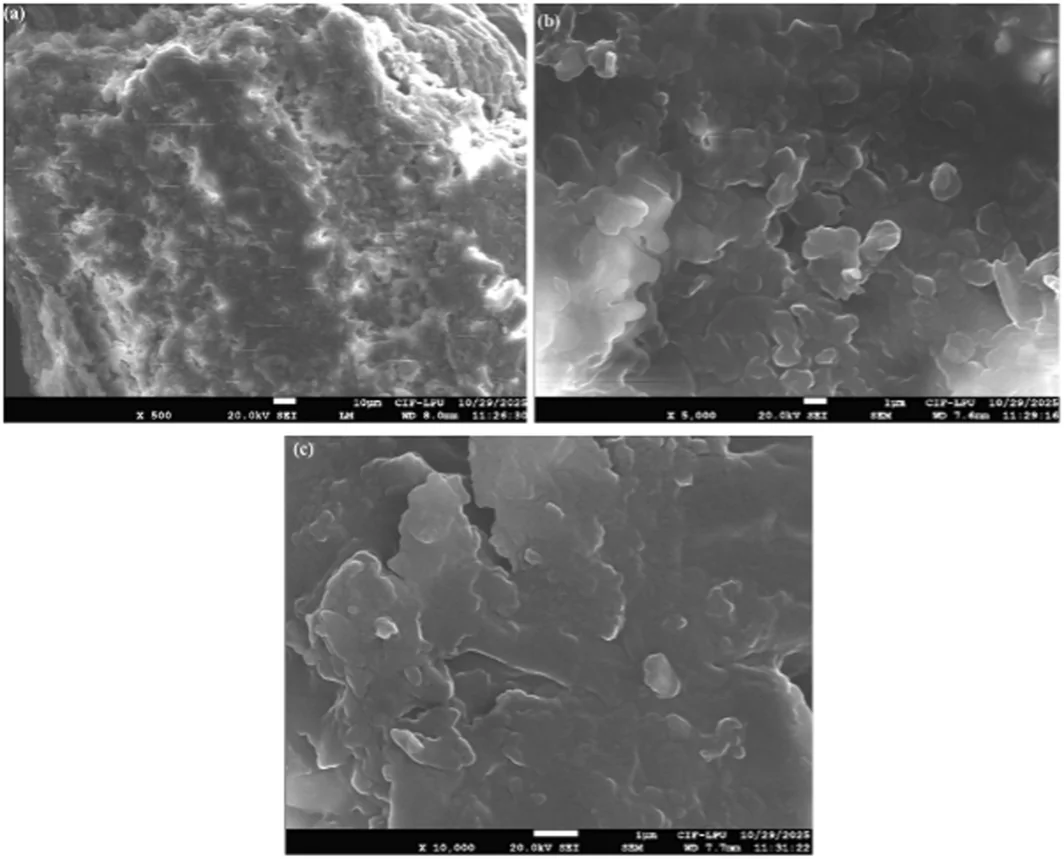
SEM micrographs of hot‐water‐blanched (1 min) banana flower at (a) 500×, (b) 5000×, and (c) 10,000× magnification, showing cell wall rupture, matrix collapse, and thermal compaction.

SEM micrographs of 1‐min hot‐water‐blanched banana flower revealed significant thermohydric disruption of tissue microarchitecture at all magnifications (Figure 5). At 500×, the surface appeared swollen, compacted, and irregular, reflecting rapid water uptake and heat‐mediated softening of the cell wall matrix. Loss of a defined fibrous network indicates a reduction in mechanical rigidity and partial collapse of cellular compartments, characteristic of parenchymatous tissue under thermal stress. At 5000×, extensive cell wall disruption, intercellular separation, and amorphous aggregation were observed, attributable to solubilization of pectins and hemicelluloses, which weakens the middle lamella and destroys cell‐to‐cell adhesion. While initial disruption transiently increases solvent access, it simultaneously promotes leaching of water‐soluble phenolics and antioxidants into the blanching medium, reducing their retention in the solid matrix. At 10,000×, ruptured lamellar structures and fused cellular debris indicated irreversible thermal damage to the polysaccharide–protein matrix. The collapse of microporosity and matrix fusion suggests structural densification rather than diffusion‐enhancing porosity, limiting extractable surface area. These SEM findings are consistent with the lower phenolic retention and antioxidant activity of hot‐water‐treated samples and confirm that 1‐min hot‐water blanching is less effective than either citric acid or microwave treatment for bioactive preservation in banana flower (Yaseen et al. 2025).

##### 3.3.1.4 SEM of Banana Flower Blanched With Microwave (40 s, 720 W)

**FIGURE 6:**
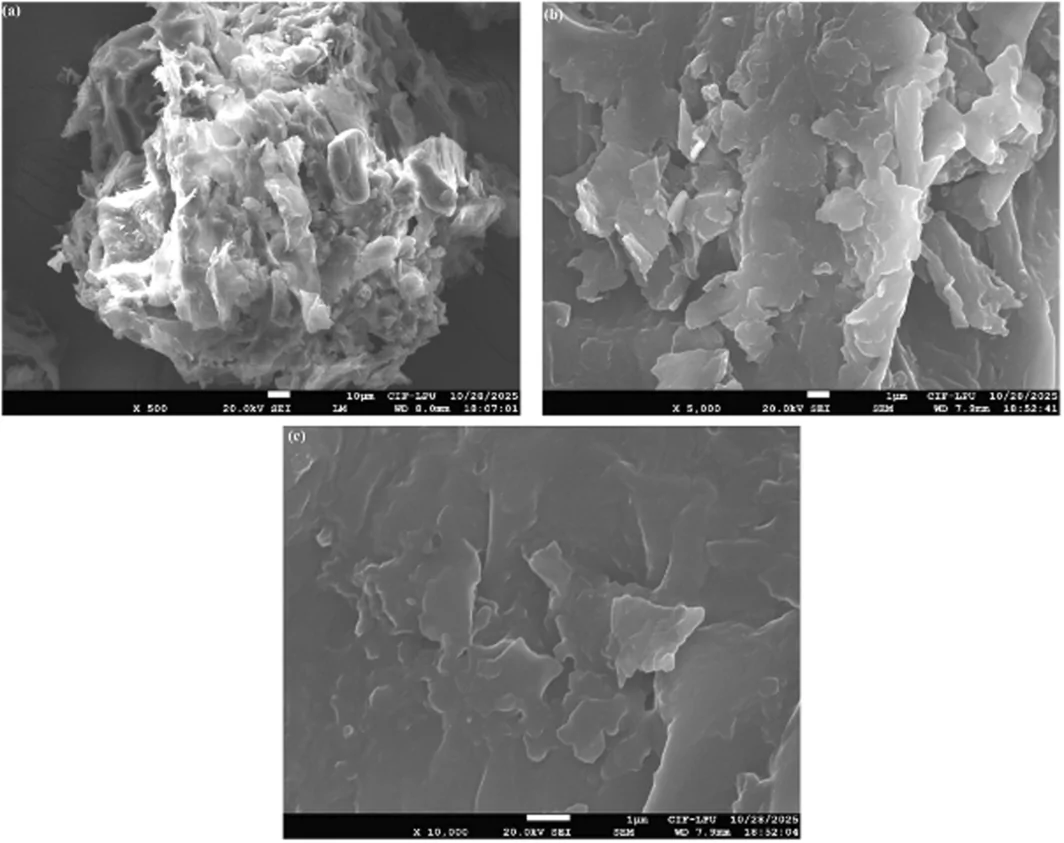
SEM micrographs of MW40‐blanched banana flower at (a) 500×, (b) 5000×, and (c) 10,000× magnification, showing severe tissue fragmentation, delaminated cell walls, and interconnected micropores.

Microwave blanching at 720 W for 40 s (MW40) induced the most extensive and extraction‐favorable microstructural modifications among all treatments evaluated (Figure 6). At 5000×, delaminated cell walls, interconnected voids, and a highly porous network were evident, with lamellar cell wall layers peeled apart to form open diffusion channels and microcavities. These features indicate disruption of pectic and hemicellulosic components that normally cement cell wall layers, dramatically increasing solvent accessibility and internal surface area for subsequent extraction. At 10,000×, extensive microfractures, exfoliated flakes, and exposed inner cell wall matrices were observed. The cellulose‐rich framework was partially preserved but heavily disrupted, with broken fibrils and detached wall fragments. This micro‐scale damage directly facilitates liberation of cell‐wall‐bound phenolics many of which are esterified to polysaccharides, converting them into more extractable forms. Overall, MW40 induced the most aggressive and extraction‐favorable structural transformation among blanching treatments tested. The combination of cell bursting, delamination, and microporosity formation creates a highly permeable matrix that facilitates rapid solvent penetration, enhanced diffusion, and improved solubilization of bioactive compounds (Rrucaj et al. 2023). This structural evidence provides a mechanistic basis for the significantly higher TPC, TFC, and antioxidant activity (DPPH, ABTS, FRAP) observed in MW40‐treated banana flower, as reported in Sections 3.2.1, 3.2.2, 3.2.3.

#### 3.3.2 X‐Ray Diffraction (XRD) Analysis

**FIGURE 7:**
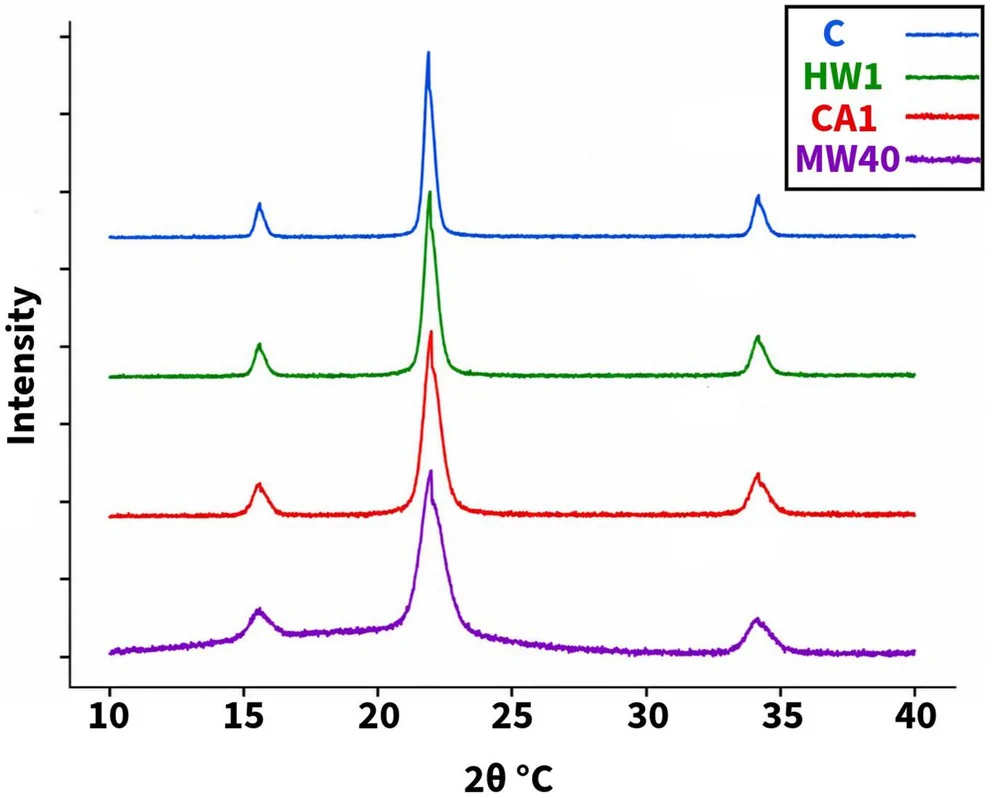
XRD patterns of banana flower powders under citric acid, hot water, and microwave blanching, showing progressive amorphization and reduction in crystalline order with increasing treatment intensity.

X‐ray diffraction analysis of banana flower powder subjected to varying blanching conditions, citric acid (CA1), hot water (HW1), and microwave (MW40) revealed characteristic diffraction peaks with treatment‐dependent differences in peak breadth and intensity (Figure 7), providing information about the effect of blanching on the supramolecular organization of cell wall polymers.

Citric‐acid‐blanched samples (CA1) exhibited diffraction peaks at 2θ = 15.6°, 21.9°, and 34.2°, corresponding to the (1–10), (200), and (004) planes of cellulose I, with slight broadening and a relative crystallinity index (CrI) of 34.5%. This moderate amorphization reflects selective acid hydrolysis of amorphous hemicellulose‐pectin matrices and loosening of cellulose microfibril packing, increasing matrix permeability without fully disrupting the ordered crystalline structure. Hot‐water‐blanched samples (HW1) showed similar cellulose I peak positions with slightly broader profiles and a CrI of ~37.8%, marginally higher than CA1. This comparatively higher crystallinity likely reflects partial recrystallization or thermal relaxation of cell wall polymers during hot‐water treatment, as well as selective dissolution of amorphous regions. The higher structural rigidity of HW1 may restrict solvent access and limit liberation of bound phenolics, contributing to its lower bioactive recovery (Lomovskiy et al. 2020).

Microwave‐blanched samples (MW40) showed the greatest structural disruption, with diffraction peaks at 15.7°, 21.8°, and 34.3° exhibiting the highest degree of peak broadening and intensity reduction, and the lowest CrI (~29.7%). A diffuse scattering hump at approximately 19.2° was observed in the MW40 pattern. While this feature has been associated with cellulose II formation in some literature contexts, cellulose I → II conversion typically requires mercerization with concentrated alkali or regeneration from solution (Seddiqi et al. 2021), and has not been widely documented under microwave blanching conditions. A more parsimonious interpretation is that the 19.2° hump reflects increased amorphous scattering from disrupted and disordered cellulose microfibrils rather than true cellulose II formation. The extensive amorphisation confirmed by the lowest CrI (~29.7%) is attributed to microwave‐induced volumetric heating and internal steam pressure generation (Kostas et al. 2017), which may disrupt hydrogen‐bonding networks and microfibrillar packing. This reduced crystallinity and increased amorphous character in MW40 may enhance enzyme access, solvent diffusion, and release of cell‐wall‐bound phenolics and flavonoids, consistent with the superior antioxidant activity observed for MW40‐treated samples.

#### 3.3.3 Differential Scanning Calorimetry (DSC) Analysis

**FIGURE 8:**
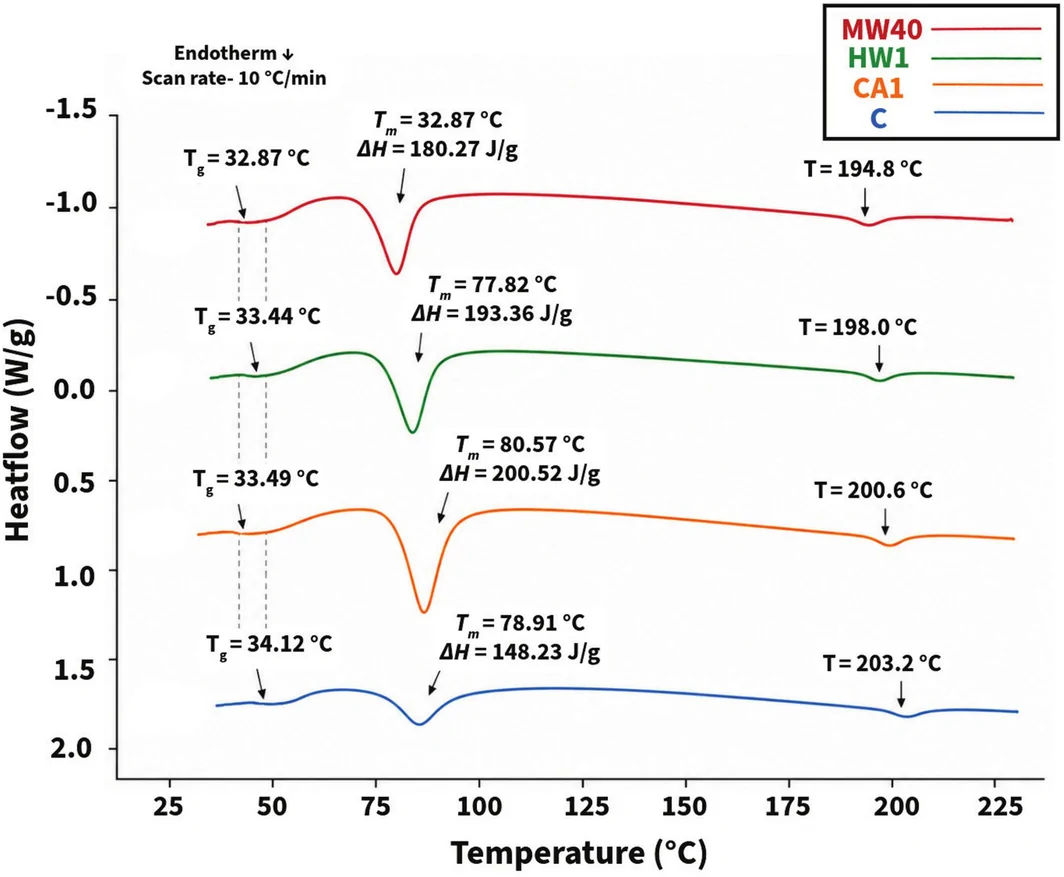
DSC thermograms of banana flower powders subjected to 1% citric acid, hot water, and microwave (720 W, 40 s) blanching treatments.

Differential scanning calorimetry thermograms for banana flower powders subjected to 1% citric acid (CA1), hot water (HW1), and microwave (MW40) blanching are presented in Figure 8. CA1 appeared to exhibit a single well‐defined endothermic peak (onset: 31.49°C; peak: 80.57°C; endset: 116.84°C) with the highest enthalpy change (ΔH = 200.52 J/g), indicating a well‐organized biopolymeric matrix in which bioactive compounds are firmly integrated into the cell wall network. The mild hydrolytic and chelating actions of citric acid may facilitate cell wall opening without disrupting polymer integrity, preserving strong phenolic–polysaccharide interactions and thermal resistance. HW1 produced two low‐enthalpy endothermic peaks (~81°C and ~198°C; ΔH = 75.18 and 0.81 J/g, respectively), which may indicate heterogeneous phase separation and structural discontinuity (Iijima et al. 2020). The low ΔH values may reflect a loss of ordered molecular interactions and matrix integrity consistent with the thermal breakdown observed in SEM analysis, explaining the impaired bioactive retention of hot‐water‐blanched samples. MW40 appeared to exhibit a single endothermic transition (onset: 32.87°C; peak: 73.64°C; endset: 116.98°C; ΔH = 180.27 J/g), indicating that despite severe microstructural disruption evidenced by SEM and XRD, the molecular biopolymer network retained substantial thermal stability. This may reflect the limited duration of microwave treatment, which achieves rapid internal heating and cell rupture without prolonged thermal degradation. Collectively, DSC analysis suggests that CA1 and MW40 preserve molecular‐level polymer integrity more effectively than HW1, with MW40 additionally offering the structural accessibility that maximizes bioactive extractability (Sun et al. 2022; Tang et al. 2024).

### 3.4 Comparative Context With Prior Literature

The findings of this study broadly align with, yet extend, the existing literature on banana flower and Musa by‐product processing. Studies of blanching effects on banana flower have reported that thermal pretreatments differentially affect phenolic retention depending on the aqueous exposure and treatment duration, a pattern consistent with the TPC and antioxidant gradients observed here across HW, MW, and CA treatments. The superior performance of shorter‐duration, low‐aqueous‐contact blanching (microwave) in phenolic preservation is supported by Saini et al. (2023), who reported similar trends in cell‐wall‐bound phenolic release under microwave heating in vegetable matrices. Studies on *Musa* peel under analogous thermal conditions have similarly reported microwave superiority for antioxidant retention (Vu et al. 2016). The CIELAB color data obtained here (ΔE range: 2.83–9.74) are consistent with reported color changes in thermally processed banana products and confirm that citric acid blanching causes the least consumer‐perceptible color deviation, a finding of practical relevance for clean‐label product development. The enzyme activity data (PPO and POD) obtained in this study provide direct mechanistic evidence that is not commonly reported in banana flower blanching literature, strengthening the interpretation that antioxidant preservation under microwave treatment is enzymatically mediated rather than purely structural.

### 3.5 Limitations

The inherent limitations of the study should be acknowledged. First, microwave blanching was conducted using a domestic microwave oven (700–720 W), which may limit power uniformity and reproducibility compared with industrial microwave systems; future studies should validate these findings using pilot‐scale microwave equipment with quantifiable energy delivery. Second, while this study measured PPO and POD activity to confirm enzyme inactivation, the specific relationship between residual enzyme activity thresholds and consumer‐relevant browning endpoints was not modeled, which warrants further investigation. Third, in vitro antioxidant assays (DPPH, ABTS, and FRAP) used here do not account for bioaccessibility or bioavailability of phenolics under gastrointestinal conditions; in vitro digestion studies would strengthen the functional food application claims. Fourth, individual polyphenol profiling by high‐performance liquid chromatography‐mass spectrometry (HPLC‐MS) was not conducted; quantitative identification of specific phenolic and flavonoid compounds would allow more precise mechanistic interpretation. Fifth, plant material was sourced from a single geographical location and season, which may limit the generalizability of proximate and phytochemical values across cultivars and growing conditions. Finally, sensory evaluation data are not reported; while CIELAB color analysis provides objective color metrics, consumer acceptability studies would be needed to translate these findings directly into product development recommendations.

## 4 Conclusion

This study systematically evaluated the effects of three blanching methods: hot‐water, citric acid, and microwave treatment on the phytochemical profile, antioxidant activity, enzyme inactivation, color characteristics, and structural properties of banana flower ( *Musa acuminata* ). Microwave blanching at 720 W for 40 s (MW40) produced the highest retention of TPC (7.60 mg GAE/g), TFC (4.90 mg QE/g), and antioxidant capacity (DPPH: 57.97; ABTS: 63.83; FRAP: 75.72 μmol TE/g), which may be attributable to rapid enzyme inactivation, minimal aqueous leaching, and the formation of an extraction‐favorable microporous cell wall architecture. Citric acid blanching (1%) achieved the most complete polyphenol oxidase (PPO) (7.74% residual) and peroxidase (POD) (12.35% residual) inactivation and produced the smallest color deviation from the control (ΔE: 2.83), making it a viable low‐heat alternative for applications where color stability is a priority. Hot‐water blanching caused the greatest losses across all bioactive parameters, with deterioration increasing with treatment duration. Future research should address the following specific directions: (1) validation of the optimized microwave blanching parameters (duration, power level) using industrial‐scale microwave equipment with monitored energy delivery to ensure process scalability and reproducibility; (2) HPLC‐MS profiling of individual phenolic and flavonoid compounds in blanched banana flower fractions to enable compound‐specific stability modeling; (3) in vitro gastrointestinal digestion and bioaccessibility studies on blanched banana flower extracts to determine the fraction of phenolics bioavailable post‐digestion; (4) incorporation of blanched banana flower powder into model food systems (e.g., bread, yogurt, and meat analogues) with sensory panel evaluation to assess consumer acceptance and functional performance under food matrix conditions; (5) multi‐cultivar and multi‐season studies across diverse Musa genotypes to establish whether the blanching treatment rankings observed here are generalizable; and (6) investigation of the interaction between blanching‐induced structural changes and in vitro protein digestibility, given the meaningful protein content (5.58%) of banana flower powder.

## Author Contributions

**Saurabh Bhosle:** conceptualization, investigation, methodology, writing – review and editing, writing – original draft, formal analysis. **Sonia Morya:** investigation, writing – original draft, writing – review and editing, supervision, data curation, validation. **Robert Mugabi:** writing – review and editing, validation, methodology.

## Funding

The authors have nothing to report.

## Conflicts of Interest

The authors declare no conflicts of interest.

## Data Availability

The data that support the findings of this study are available from the corresponding author upon reasonable request.
